# Molecular Mechanisms of Parathyroid Disorders in Chronic Kidney Disease

**DOI:** 10.3390/metabo12020111

**Published:** 2022-01-25

**Authors:** Alia Hassan, Nareman Khalaily, Rachel Kilav-Levin, Morris Nechama, Oded Volovelsky, Justin Silver, Tally Naveh-Many

**Affiliations:** 1 Minerva Center for Bone and Mineral Research, Nephrology Services, Hadassah—Hebrew University Medical Center, Jerusalem 91120, Israel; aliahassan811@gmail.com (A.H.); nareman.khalaily@mail.huji.ac.il (N.K.); erlevin@gmail.com (R.K.-L.); silver@mail.huji.ac.il (J.S.); 2 Nursing, Jerusalem College of Technology, Jerusalem 91160, Israel; 3 Pediatric Nephrology, Hadassah—Hebrew University Medical Center, Jerusalem 91120, Israel; mnehama@gmail.com (M.N.); odedvo@hadassah.org.il (O.V.); 4 The Wohl Institute for Translational Medicine, Hadassah—Hebrew University Medical Center, Jerusalem 91120, Israel

**Keywords:** parathyroid hormone, secondary hyperparathyroidism, post-transcriptional regulation, parathyroid cell proliferation, microRNA

## Abstract

Secondary hyperparathyroidism (SHP) is a common complication of chronic kidney disease (CKD) that induces morbidity and mortality in patients. How CKD stimulates the parathyroid to increase parathyroid hormone (PTH) secretion, gene expression and cell proliferation remains an open question. In experimental SHP, the increased *PTH* gene expression is post-transcriptional and mediated by PTH mRNA–protein interactions that promote PTH mRNA stability. These interactions are orchestrated by the isomerase Pin1. Pin1 participates in conformational change-based regulation of target proteins, including mRNA-binding proteins. In SHP, Pin1 isomerase activity is decreased, and thus, the Pin1 target and PTH mRNA destabilizing protein KSRP fails to bind PTH mRNA, increasing PTH mRNA stability and levels. An additional level of post-transcriptional regulation is mediated by microRNA (miRNA). Mice with parathyroid-specific knockout of Dicer, which facilitates the final step in miRNA maturation, lack parathyroid miRNAs but have normal PTH and calcium levels. Surprisingly, these mice fail to increase serum PTH in response to hypocalcemia or uremia, indicating a role for miRNAs in parathyroid stimulation. SHP often leads to parathyroid hyperplasia. Reduced expressions of parathyroid regulating receptors, activation of transforming growth factor α-epidermal growth factor receptor, cyclooxygenase 2-prostaglandin E2 and mTOR signaling all contribute to the enhanced parathyroid cell proliferation. Inhibition of mTOR by rapamycin prevents and corrects the increased parathyroid cell proliferation of SHP. This review summarizes the current knowledge on the mechanisms that stimulate the parathyroid cell at multiple levels in SHP.

## 1. Introduction

Parathyroid hormone (PTH) regulates serum calcium and phosphate levels and bone strength. A reduction in serum calcium stimulates PTH secretion and, when prolonged, parathyroid cell proliferation [1,2,3]. A 7-transmembrane G-protein-coupled calcium-sensing receptor (CaSR) senses changes in extracellular serum calcium. Increased calcium activates the parathyroid CaSR, leading to decreased PTH secretion [4,5,6]. Other factors that act on the parathyroid are serum phosphate that stimulates and vitamin D and the bone-derived phosphaturic hormone fibroblast growth factor 23 (FGF23) that suppress PTH synthesis, secretion and proliferation [7,8,9,10]. A recent study suggested that the CaSR also acts as a phosphate sensor in the parathyroid gland. According to this study, phosphate increases PTH secretion by inhibiting the CaSR in a noncompetitive manner [11]. The active metabolite of vitamin D, 1,25-dihydroxy-vitamin D (1,25D), suppresses *PTH* gene transcription. It binds to the vitamin D receptor (VDR), which heterodimerizes with retinoic acid X receptors. The complex then enters the nucleus to bind vitamin D-response elements in the PTH gene promoter region [12,13,14,15]. FGF23 acts on the FGFR1-klotho receptor complex, expressed in the parathyroid, to decrease PTH expression [9,16]. Secondary hyperparathyroidism (SHP) is a common complication of chronic kidney disease (CKD) that correlates with morbidity and mortality in these patients. SHP is characterized by increased *PTH* gene expression and secretion and parathyroid gland hyperplasia [17,18,19,20]. Many factors combine to form SHP in CKD. These include phosphate retention, hyperphosphatemia, hypocalcemia and low levels of 1,25D. In addition, parathyroid gland hyperplasia leads to reduced expression of the VDR, CaSR and FGF23 receptor complex in experimental SHP models and human parathyroid samples [3,19,21]. Other parathyroid post-receptor mechanisms also affect PTH expression and parathyroid cell proliferation. The magnitude of SHP is determined by two major mechanisms, an increase in PTH synthesis and an increase in parathyroid gland mass [22]. Parathyroid cells have a limited number of secretory granules containing preformed hormone compared to other endocrine cells. Therefore, increases in *PTH* gene expression are essential for a continued secretion of PTH. The increased *PTH* gene expression in experimental SHP induced by either uremia or prolonged hypocalcemia is due to post-transcriptional mechanisms that alter PTH mRNA stability and levels [23,24,25,26].

## 2. Post-Transcriptional Mechanisms Regulating Parathyroid Hormone Gene Expression in Secondary Hyperparathyroidism

### 2.1. Regulation of PTH mRNA Stability by Protein–PTH mRNA Interactions and Pin1

The balance between the rates of gene transcription and mRNA decay determines cytoplasmic levels of all mRNAs. mRNA decay is tightly regulated by mRNA-binding proteins that bind to specific sequences in mRNAs [27,28,29]. In most mRNAs, these sequences are adenosine-uridine (AU)-rich elements (AREs) embedded in the 3′-untranslated region (UTR) [28,30,31,32,33,34]. AU-rich binding factor 1 (AUF1, *HNRNPD* gene product) and K-homology splicing regulatory protein (KSRP) are two mRNA-binding proteins that bind ARE containing mRNA 3′-UTRs [28,30,31,35]. AUF1 promotes either decay or stability, depending on the cell type and mRNA [36,37]. KSRP induces mRNA decay by recruiting the multi-subunit RNA ribonuclease complex, exosome to the mRNA 3′ ends [38]. Post-translational modifications of the mRNA-binding proteins affect protein–mRNA interactions [35,39,40,41,42,43]. Post-transcriptional regulatory mechanisms of thr mRNA binding proteins contribute to the regulation of gene expression in a wide variety of cellular and physiological processes, including hormones. Insulin and insulin-like growth factor expression, as well as the expression of different reproductive hormones is regulated by gene transcription as well as changes in mRNA stability, localization and mRNA translation efficiency [44,45,46,47]. Post-transcriptional mechanisms also mediate the increased *PTH* gene expression by hypocalcemia and uremia in SHP [48].

**Figure 1:**
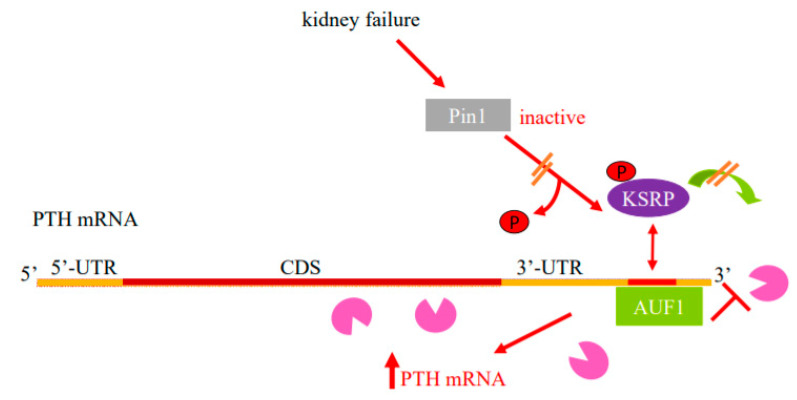
Model for the regulation of PTH mRNA stability in SHP by protein–PTH mRNA interactions and Pin1. Basal PTH mRNA levels are determined by a balanced interaction of the PTH mRNA 3′-UTR ARE with its stabilizing protein AUF1 and destabilizing protein KSRP that recruits the exosome (Pac-Man) to mRNAs. The isomerase Pin1 binds KSRP and induces KSRP Ser181 conformational change and dephosphorylation, which increases KSRP-PTH mRNA interaction and mRNA decay. In SHP, due to kidney failure or hypocalcemia, there is decreased parathyroid Pin1 isomerase activity and phosphorylated KSRP fails to bind PTH mRNA and recruit the exosome to it. As a result, AUF1 binds PTH mRNA with greater affinity, leading to increased PTH mRNA stability and levels. Schematic representation of the PTH mRNA with the coding sequence (CDS) in brown, the 5′- and 3′-untranslated regions (UTRs) in orange and ARE in red. AUF1, AU-rich binding factor 1; KSRP, K-homology splicing regulatory protein. Adapted with permission from the FASEB Journal [26].

**Figure 2:**
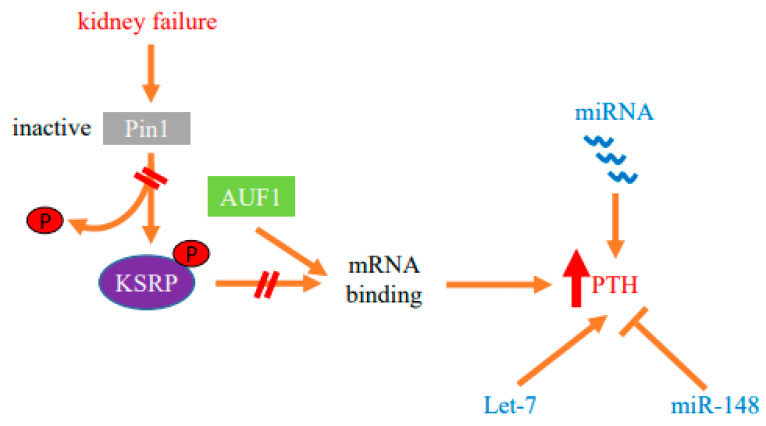
Protein–mRNA interactions and miRNA determine the post-transcriptional increase in parathyroid hormone (*PTH*) gene expression in SHP. Decreased activity of the isomerase Pin1 in the parathyroid in SHP modifies protein–PTH mRNA interactions to increase PTH mRNA stability and levels in experimental chronic kidney failure. The stimulation of PTH expression is also dependent upon miRNA. Parathyroid miRNAs are dysregulated in both chronic kidney disease patients and experimental models. Mice with parathyroid specific knockout of Dicer, which prevents global miRNA maturation and function, fail to increase serum PTH after acute and chronic hypocalcemia or uremia, demonstrating that miRNAs are essential for parathyroid stimulation in SHP. Let-7 and miR-148 miRNA families that are dysregulated in CKD-SHP modify PTH secretion in vivo and in vitro, suggesting roles for these miRNAs in the increased PTH expression.

Enhanced PTH mRNA stability is the major mechanism that increases *PTH* gene expression in experimental SHP due to either dietary induced prolonged hypocalcemia or uremia. Increased PTH mRNA binding to stabilizing AUF1 and decreased binding to the decay-promoting protein KSRP shifts the balance towards increased PTH mRNA stability and levels [23,24,25]. These proteins bind to an evolutionary conserved ARE in the PTH mRNA 3′-UTR [49] (Figure 1). The peptidyl prolyl *cis/trans* isomerase Pin1 coordinates the interaction of these ARE binding proteins to the PTH mRNA. Pin1 specifically binds phosphorylated serine/threonine-proline motifs in target proteins and induces conformational changes of the peptidyl bond [50,51]. By inducing conformational changes, Pin1 controls the activity of many phosphoproteins in a wide range of cellular activities [52,53]. Pin1 also binds RNA-binding proteins, including AUF1 and KSRP, to regulate the stability of ARE containing mRNAs that include GM-CSF, TGF-β1 and PTH [53,54,55,56]. During oocyte maturation, Pin1 binds phosphorylated cytoplasmic polyadenylation element binding (CPEB) protein that regulates mRNA translation through cytoplasmic polyadenylation. This leads to CPEB isomerization and subsequent degradation in a ubiquitination-dependent manner [57,58]. In the parathyroid, Pin1 induces PTH mRNA decay by altering KSRP phosphorylation and KSRP-PTH mRNA binding [54,59]. Pin1-KSRP interaction leads to Pin-dependent KSRP conformational change that leads to dephosphorylation of KSRP at Ser181. Un-phosphorylated KSRP then binds PTH mRNA with higher affinity, inducing PTH mRNA decay. This regulation of PTH mRNA stability and levels by Pin1 is dependent upon the PTH mRNA 3′-UTR ARE. In SHP, Pin1 isomerase activity is decreased and phosphorylated KSRP fails to bind PTH mRNA, resulting in increased PTH mRNA and serum levels. In accordance, pharmacologic inhibition of Pin1 increased PTH mRNA levels post-transcriptionally in vivo, in rat parathyroid glands in culture and in transfected cells. Furthermore, *Pin1^−/−^* mice have increased serum PTH and PTH mRNA levels [54]. Therefore, Pin1 decreased activity, parathyroid Pin1-KSRP interaction and KSRP-PTH mRNA binding play a significant role in the regulation of PTH mRNA stability and in the pathogenesis of the SHP (Figure 1 and Figure 2).

Recently, mRNA profiles of porcine parathyroid glands were performed in a long-term dietary phosphorus intervention by keeping pig offspring on distinct mineral dietary phosphorus levels throughout fetal and postnatal life. RNA sequencing data and the resulting molecular pathways of parathyroid glands showed that PTH abundance is controlled via Pin1, CaSR, MAfB, Phospholipase C and proteinase A signaling to regulate *PTH* gene expression, stability and secretion. Parathyroid glands revealed lowered *Pin1* mRNA abundance in animals fed a low phosphorus diet with no change in the expression of *KSRP* by post-weaning diets [60].

In patients with diffuse and nodular hyperparathyroidism, normal and hyperplastic human parathyroid tissue microarrays showed that AUF1 was among the differentially expressed genes [61]. We analyzed the publicly deposited mRNA profiling data from a study comparing adenomas to normal human parathyroid tissues (NCBI Gene Expression Omnibus accession number GSE83421) [62]. We found that *Pin1* mRNA was 30% higher (*p* < 0.01) in parathyroid adenomas vs. normal parathyroid tissues. Additional proteins that were related to RNA stability as well as protein synthesis and processing, DNA repair and cell growth were also dysregulated [26]. In another human study of single nucleotide polymorphisms (SNP) in the *Pin1* gene promoter, in the Chinese Han population in Northwest China, Pin1 C667T genetic variants were associated with CKD SHP. They suggested that the Pin1 variant genotypes may be used as biomarkers for susceptibility to CKD SHP [63]. Together, these studies support a role for Pin1 and PTH mRNA interacting proteins not only in experimental models, but also in human SHP patients. 

### 2.2. Parathyroid microRNA Ablation in Experimental Secondary Hyperparathyroidism

MicroRNAs (miRNAs) provide an additional post-transcriptional mechanism of gene expression. miRNAs are short endogenous non-coding RNAs that affect gene expression by inducing sequence-specific mRNA decay and/or translation repression of target mRNAs [64,65]. miRNAs are transcribed as primary (pri)-miRNA transcripts that are first cleaved by nuclear Drosha and then by Dicer in the cytoplasm to generate the mature miRNA [66]. Hence, Dicer is essential for the formation of mature and functional miRNAs. Mice deficient in *Dicer* die at embryonic day E8.5 [67]. However, tissue-specific *Dicer* knockout mice are useful to study the role of miRNAs in a particular tissue [68,69,70]. To study the role of miRNAs in the parathyroid, we generated mice with parathyroid-specific gene deletion of *Dicer* (PT-*Dicer^−/−^* mice). The PT-*Dicer^−/−^* mice developed normally and had normal basal serum PTH, calcium and phosphate levels. Remarkably, the PT-*Dicer^−/−^* mice failed to increase serum PTH after the stimuli of acute and chronic hypocalcemia. Moreover, the PT-*Dicer^−/−^* mice also failed to increase serum PTH levels when stressed by an adenine high phosphorus diet to induce CKD [48,71]. Therefore, intact Dicer and miRNAs are essential for activation of the parathyroid by the major stimuli for PTH secretion, acute and chronic hypocalcemia and uremia (Figure 2).

### 2.3. Parathyroid miRNA Profiles

Differentially expressed miRNAs have been described in primary hyperparathyroidism of adenomas and carcinoma patients, compared to normal parathyroid tissue [72,73,74,75,76]. Studies in parathyroid carcinomas identified miRNAs that were downregulated when compared to controls, and are common to other human cancers [77,78]. Interestingly, parathyroid carcinomas showed deregulation of miRNAs belonging to the largest human chromosome 19 miRNA cluster [79]. Downregulation of miR-296-5p was the best predictor in differentiating parathyroid carcinomas from normal parathyroids [80].

In contrast to parathyroid carcinoma and adenoma, parathyroid miRNA profiles of SHP where less studied. We performed the first study of miRNA expression and function in normal parathyroid physiology and in SHP, by applying small RNA sequencing, providing quantitative global miRNA profiling. We compared parathyroid tissue from control and end-stage renal disease SHP patients, as well as experimental SHP models. Of interest, there was similar miRNA content in the 50 most frequent miRNA families of normal parathyroid glands from humans and rodents. Let-7 miRNA family members were the most highly expressed miRNAs in human, mouse and rat parathyroids, followed by miR-30 and miR-141/miR-200 family members [81]. SHP correlated with substantial miRNA transcriptome (miRNome) alterations. miRNA levels were substantially dysregulated by miRNA sequencing in both hyperplastic parathyroid glands from ESRD patients and parathyroid glands from rats fed an adenine high-phosphorus diet to induce CKD and SHP, indicating that miRNAs may be involved in the pathogenesis of CKD-SHP. Analysis of the highest expressed dysregulated miRNAs in adenine high phosphorus-induced SHP rats showed a common pattern, in which a miRNA sequence family is either upregulated or downregulated in early renal failure and the trend is increased in late CKD. miR-141 and miR-148 members both increased, peaking gradually by 8 weeks of the adenine diet-induced CKD [81]. The conservation through evolution of miRNA expression in normal parathyroids and the dysregulation of miRNAs in SHP in man, mouse and rat suggests their functional and developmental importance in SHP.

### 2.4. Function of Individual miRNAs in PTH Expression

We used anti-miR oligonucleotides, that compete with cellular target mRNAs for the binding of endogenous miRNA, to study the role of specific miRNAs in SHP [82]. We chose to inhibit let-7 members, as these are the most abundant miRNA molecules in the parathyroid and individual let-7 members were significantly dysregulated in both rat and human SHP [81]. Anti-let-7 oligonucleotides nearly doubled serum PTH levels in normal rats, and worsened SHP in uremic rats, compared to scrambled control oligonucleotides. The increased serum PTH levels observed by let-7 inhibition did not affect serum phosphate, calcium or creatinine in normal and uremic rats, suggesting a direct effect of the anti-miRs on PTH levels. Let-7 anti-miRs also increased PTH secretion in vitro, when added to normal mouse thyro-parathyroid glands in culture, confirming a primary effect of let-7 antagonism on PTH production and/or secretion in vivo in control and uremic rats and in vitro [81].

We also antagonized miR-148 and miR-141 because of the high abundance of these miRNAs in human, rat and mouse parathyroid glands and their increase in SHP. Anti miR-141 had no effect on PTH expression. However, anti miR-148 significantly decreased serum PTH levels in uremic rats, again with no change in serum creatinine or phosphate levels. Adding anti-miR-148 to thyro-parathyroid tissue from uremic mice, compared with control oligonucleotides, also decreased PTH secretion in vitro, demonstrating a direct effect of antagonizing miR-148 on the parathyroid [81]. Therefore, specific miRNA families are dysregulated in CKD-SHP. Inhibition of let-7 and miR-148 modifies PTH secretion in vivo and in vitro, suggesting roles for these miRNAs in PTH expression (Figure 2).

## 3. Parathyroid Cell Proliferation in Secondary Hyperparathyroidism

**Figure 3:**
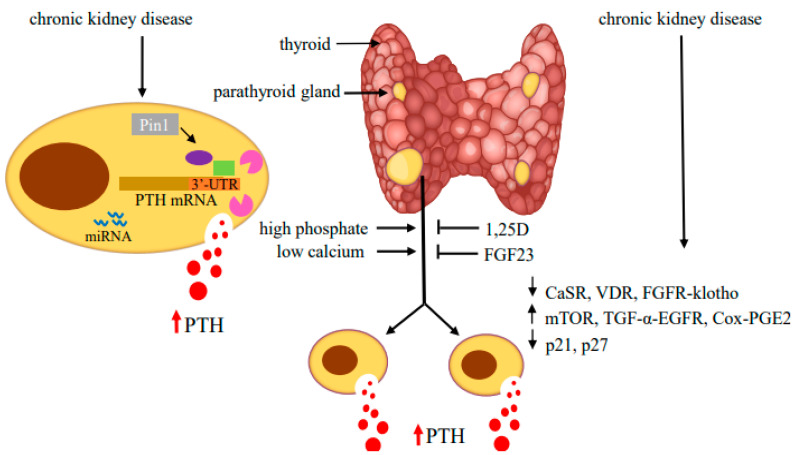
Regulators of PTH gene expression and parathyroid cell proliferation in SHP. Hyperphosphatemia and hypocalcemia stimulate and 1,25(OH)_2_ vitamin D (1,25D) and fibroblast growth factor (FGF) 23 inhibit PTH production and parathyroid cell proliferation. In CKD-induced SHP, decreased expression of the CaSR, VDR and FGFR-klotho receptors together with activation of mTOR, TGF-α-EGFR and Cox-PGE2 signaling and changes in cell cycle regulators lead to hyperplastic parathyroid glands with high serum PTH levels. In the parathyroid cell, protein–PTH mRNA interactions mediated by activity of the isomerase Pin1 and miRNA increase PTH production and secretion. Adapted with permission from the International Journal of Molecular Sciences [99].

The increase in *PTH* gene expression is tightly linked to increased parathyroid mass due to parathyroid cell proliferation and, to a lesser extent, an increase in cell size. The molecular pathways mediating parathyroid cell proliferation are still unclear. The parathyroid cells are generally quiescent under physiological conditions, with a low turnover rate and mitoses [83]. However, the cells retain their potential to proliferate in response to uremia, hypocalcemia, hyperphosphatemia and vitamin D deficiency [84]. In CKD patients, at the early stages of SHP, the parathyroid glands show diffuse and polyclonal proliferation. Prolonged uremia drives the progression of parathyroid cell proliferation from diffuse to nodular growth [85,86]. The main factors responsible for parathyroid hyperplasia are similar to those that increase PTH biosynthesis and secretion. High phosphate, hypocalcemia and 1,25D deficiency in CKD all contribute to the development of hyperplasia [87].The studies on parathyroid cell proliferation in experimental SHP of renal failure mainly rely on either 5/6 nephrectomy performed by removing one kidney and 2/3 of the contralateral kidney or an adenine diet. SHP is further increased when the rodents in these models are fed a high phosphorus diet [7,17,18,88,89,90,91]. In both experimental models and in patients, the downregulation of CaSR and VDR expression is associated with parathyroid cell proliferation. The decrease in parathyroid CaSR occurs in CKD only when the parathyroid glands are hyperplastic, and may contribute to the increased parathyroid proliferation in SHP [92,93,94,95,96]. Phosphate restriction prevents both parathyroid hyperplasia and the decreased CaSR expression in CKD. Uremic patients with SHP, as well as rodents with experimental SHP, have decreased VDR mRNA and protein levels [21,93,97,98]. These and other studies suggested that parathyroid gland hyperplasia reduces expression of both the CaSR and VDR, which further contributes to the high PTH secretion and parathyroid cell proliferation in SHP (Figure 3).

The classical treatment for SHP patients included active vitamin D compounds and phosphate binders, to limit gastrointestinal phosphate absorption [100]. Administration of vitamin D to rats with secondary hyperparathyroidism reduced parathyroid cell proliferation and increased both VDR and CaSR expression [17,101,102]. However, while vitamin D compounds suppress PTH secretion, they also promote calcium and phosphate intestinal absorption. Calcimimetic compounds are positive allosteric modulators of the CaSR that increase the sensitivity of the CaSR, thereby decreasing PTH secretion. Calcimimetics lower PTH levels in CKD patients with SHP. Studies using experimental CKD rat models have demonstrated that calcimimetics reduce parathyroid hyperplasia, as measured by gland weight, DNA content and staining for proliferation markers [103]. Reduction in parathyroid gland volume has also been demonstrated using imaging studies in dialysis patients receiving calcimimetic [94,104]. Calcimimetics increase cell surface CaSR and VDR expression in the parathyroid cells in uremic rats [105]. The calcimimetic R568 also reduced *PTH* gene expression in CKD rats by decreasing PTH mRNA stability through increased parathyroid KSRP–PTH mRNA interactions compared to untreated CKD rats [106].

### 3.1. Cell Cycle Regulation

Two particular genes have been implicated in the pathogenesis of parathyroid tumorigenesis: the *cyclin D1/PRAD1* (*Cyclin parathyroid adenomatosis 1*) oncogene and the *MEN1* (multiple endocrine neoplasia type 1) tumor-suppressor gene [107,108]. Menin, the product of the *MEN1* gene, is a tumor suppressor protein in a variety of cancer types. Inactivating *MEN1* mutations lead to the development of parathyroid neoplasia in almost all patients with MEN1 [109]. PRAD1, later identified as cyclin D1, plays a vital role in controlling the cell cycle [110]. Cyclin D1 protein overexpression occurs in 20–40% of parathyroid adenomas, suggesting that increased PRAD1/cyclin D1 is one of the genetic abnormalities responsible for tumorigenesis in sporadic primary parathyroid adenomas, contributing to parathyroid hyperplasia in humans [110,111,112]. In about 8% of cases, there is DNA rearrangement involving the cyclin D encoding *CCND1* gene locus [113]. Transgenic mice with parathyroid-targeted overexpression of *cyclin D1*, modeling the gene rearrangement found in human tumors, showed that a primary defect in this cell-cycle regulator caused primary hyperparathyroidism, as in human patients [114,115]. These findings suggest that overexpression of the *cyclin D1* oncogene drives excessive parathyroid cell proliferation.

The roles of mutations in cyclin D1 and MEN1 in SHP are not entirely understood. The levels of *cyclin D1* and *retinoblastoma* gene products increase in CKD-induced hyperplastic parathyroid cells [116]. The increase in cell cycle progression in SHP correlates with a decrease in the expression of the cyclin-dependent kinase inhibitors, p21 and p27 (Figure 3). Loss of expression or function of p21 and p27 is implicated in many human malignancies [117]. The decrease in parathyroid p21 and p27 levels in SHP are most evident in cells with nodular hyperplasia. Low dietary phosphorus reduced parathyroid cell proliferation in SHP rats, together with induction of p21, and reduced expression of transforming growth factor (TGF)-α expression. A high phosphorous intake, with subsequent stimulation of parathyroid cell proliferation in 5/6 nephrectomized rats, increased TGF-α [118]. 1,25D, which prevented parathyroid gland hyperplasia in uremic rats, enhanced parathyroid p21 expression and prevented the high phosphorus-induced increase in parathyroid TGF-α content [119]. In addition, 1,25D alters membrane trafficking of the epithelial growth factor receptor (EGFR), which binds both EGF and TGF-α and downregulates EGFR-mediated growth signaling [120].

### 3.2. TGF-α and EGFR

TGF-α and its receptor EGFR are increased in uremic rats and human hyperplastic and adenomatous parathyroid glands correlating with parathyroid cell proliferation [121,122] (Figure 3). EGFR signals through MAPK activation, which in turn induces cyclin D1 and drives the cell cycle from G1 to S [122,123,124]. EGFR activation by TGF-α also reduces VDR expression and plays an important role in growth of the parathyroid gland. Inhibition of parathyroid TGF-α/EGFR prevents not only parathyroid cell proliferation, but also the reduction in VDR levels in parathyroid cells. In transgenic mice, a parathyroid-specific dominant-negative EGFR prevented the increase in serum PTH, parathyroid gland size and the decreased VDR expression observed in wild-type uremic mice [125]. EGFR also activates the AKT-mTORC1 (mammalian target of rapamycin complex 1) pathway [126,127].

### 3.3. The mTORC1 Pathway

mTOR integrates signaling pathways to regulate cell growth and proliferation. mTOR is part of mTORC1 and mTORC2. mTORC1 senses extracellular nutritional and growth factors necessary for cell growth and proliferation [128]. Rapamycin inhibits mTORC1 and cell proliferation. Different stimuli activate mTORC1 through Akt phosphorylation [129]. 4E binding protein 1 and the ribosomal protein S6 kinase 1 (S6K1) are mTORC1 targets. Upon activation, S6K1 phosphorylates its downstream target ribosomal protein S6 (rpS6) on a cluster of five serine residues at the carboxy terminus [130,131]. Knock-in mice encoding a mutant rpS6 harboring alanine substitutions at all serine phosphorylation sites (*rpS6p^−/−^* mice) have reduced body size, glucose intolerance and muscle weakness and impaired renal hypertrophy after uni-nephrectomy [132,133]. We have shown that the mTOR pathway is activated in the parathyroids of rats and mice with SHP induced by either chronic hypocalcemia or uremia. The increased mTOR activity correlated with increased parathyroid cell proliferation (Figure 3). Inhibition of mTORC1 by rapamycin both prevented and decreased parathyroid cell proliferation in SHP rats and in vitro in uremic rat parathyroid glands in organ culture. Knock-in *rpS6p^−/−^* mice had a muted increase in PTH secretion after induction of experimental CKD by an adenine high phosphorus diet and no increase in parathyroid cell proliferation, compared with the expected increases in uremic wild-type mice [134]. These results highlight the essential role of mTORC1 activation by rpS6 phosphorylation in parathyroid cell proliferation and in the pathogenesis of SHP. EGFR activates the AKT-mTORC1 pathway; therefore, the mTORC1 and EGFR pathways may act together to stimulate parathyroid cell proliferation in SHP [99] (Figure 3).

### 3.4. Nuclear Factor-Kappa B

As described above, reduced circulating 1,25D and parathyroid VDR expression play a key role in the progression of parathyroid hyperplasia. 1,25D administration partially restores parathyroid VDR expression and suppresses parathyroid cell proliferation by binding to its receptor [102]. In renal tubular cells, VDR binds to nuclear factor kappa B (NF-kB) dimers to suppress NF-κB-mediated gene transcription [135]. NF-κB is a ubiquitous transcription factor that plays a crucial role in immune and inflammatory responses. It consists of the DNA binding subunit p50 and the transactivation subunit p65/RelA (p65) [136]. In most cells, NF-kB is sequestered in the cytoplasm bound to its biological inhibitor IkB. A variety of stimuli, such as cytokines, toxins and mitogens, activate the IkB kinase complex leading to the degradation of phosphorylated IkB and the release and nuclear translocation of NF-kB [137]. Cyclin D1 is potently activated by p65 [138]. The tumor suppressor gene *menin* interacts with NF-kB and inhibits the NF-kB-mediated transcriptional activation [139]. NF-κB contributes to the pathogenesis of primary parathyroid hyperplasia [140]. In CKD-induced SHP, there was activation of the NF-κB pathway in the nodular hyperplastic parathyroid glands from patients on dialysis, with a significant increase in diffuse hyperplasia glands [141]. The reduction in VDR could lead to activation of the NF-κB pathway. In 5/6 nephrectomized rats fed a high-phosphorus diet, proliferation was higher together with activation of the NF-κB pathway, and VDR levels were decreased as expected, compared with the sham group. 1,25D decreased serum PTH and parathyroid cell proliferation, together with reduced activation of the NF-κB pathway [141]. These findings suggest that NF-κB contributes to parathyroid gland hyperplasia progression in SHP. Decreased 1,25D and VDR expression may affect parathyroid gland hyperplasia through the activation of the NF-κB pathway.

### 3.5. Cyclooxygenase 2-PGE2

Cyclooxygenase (COX) catalyzes the rate-limiting step of prostaglandin synthesis from arachidonic acid. Cox1 is constitutively expressed, while COX2 is induced by various mitogenic and inflammatory stimuli [142]. COX2 plays a significant role in carcinogenesis by promoting cell proliferation, inhibiting apoptosis and stimulating angiogenesis. COX2 is expressed in parathyroid cells of normal, hyperplastic and adenomatous parathyroid glands [143]. In end-stage renal disease patients with advanced SHP, there was enhanced expression of both COX2 and its downstream metabolic product prostaglandin E2 (PGE2) in their parathyroids [144]. Increased COX2 expression was also reported in parathyroids of 5/6-nephrectomized rats fed a high-phosphorus diet, together with the high PTH levels, parathyroid size and cell proliferation, compared to controls. Addition of PGE2 increased PTH production in dispersed primary human and bovine parathyroid cells [145,146]. In primary cultures of parathyroid glands isolated from end-stage renal disease patients, high phosphate increased PTH secretion, parathyroid cell proliferation and COX2 activity. Inhibitors of the PGE2 receptor 2 subtype (EP2) attenuated hyperparathyroidism induced by high phosphate. PGE2 or EP2 agonist directly stimulated hyperparathyroidism, suggesting that COX2 downstream PGE2 and its receptor EP2 play a role in the high phosphate-induced parathyroid hyperplasia of SHP [147] (Figure 3).

### 3.6. The Parathyroid Molecular Circadian Clock

Circadian rhythms act in metabolism, hormone secretion, cell cycle and locomotor activity. They are regulated by the molecular circadian clock, with the master clock in the suprachiasmatic nucleus of the central nervous system. In addition, internal clocks are expressed in several peripheral tissues. PTH secretion exhibits a diurnal variation and *PTH* gene promoter contains an E-box-like element in both humans and rodents, that is a target of circadian clock proteins [148,149]. A recent study by Egstrand and colleagues describes an internal operating molecular circadian clock in the parathyroid gland, which is disturbed in chronic kidney disease [150]. Because disturbed circadian rhythm may lead to abnormal growth, Egstrand and colleagues studied the expression of parathyroid clock and clock-regulated cell cycle genes in parathyroid glands of normal and CKD rats. They found that circadian clock genes were rhythmically expressed in normal parathyroid glands. In particular, they showed significant rhythmicity of parathyroid FGFR1, MafB and Gata3 transcription factors. Importantly, in CKD rats, there was deregulation of the parathyroid circadian clock genes and the cell cycle regulators Cyclin D1, c-Myc, Wee1 and p27, which are all influenced by the circadian clock [150]. Thus, a circadian clock operates in the parathyroid and its downstream cell cycle regulators are altered in CKD. The parathyroid circadian clock may contribute to parathyroid cell proliferation in SHP.

## 4. Summary

SHP is a progressive disease occurring in most CKD patients, with severe consequences for patient health. If poorly controlled, SHP progresses and leads to bone disease and soft tissue calcification, which influence morbidity and mortality. SHP is characterized by increased PTH secretion, gene expression and parathyroid cell proliferation. Studies in experimental SHP show that the high *PTH* gene expression in SHP is determined by post-transcriptional mechanisms mediated by protein binding to the PTH mRNA 3′-UTR. Pin1 coordinates these interactions. Decreased parathyroid Pin1 enzymatic activity in SHP alters the PTH mRNA–protein interaction, leading to increased PTH mRNA stability and levels. miRNAs provide an additional layer of post-transcriptional regulation of gene expression. Parathyroid-specific deletion of the miRNA maturating enzyme Dicer in PT-*Dicer^−/−^* mice results in a muted increase in PTH expression by both hypocalcemia and uremia, indicating that parathyroid miRNAs are crucial for the stimulation of the parathyroid in SHP. Profiling of the human, mouse and rat parathyroid miRNome by small-RNA sequencing showed that human and rodent parathyroids share similar miRNA profiles. SHP leads to the dysregulation of some of these, as well as other miRNAs. The evolutionary conservation of abundant miRNAs in normal parathyroids and the regulation of miRNA expression in SHP indicate their significance in parathyroid function and in the development of hyperparathyroidism. In addition to the stimulated PTH production per cell, increased parathyroid cell proliferation also contributes to SHP. The physiologic regulation of parathyroid cell hyperplasia by calcium, phosphate, 1,25D and FGF23 is interrupted in advanced CKD and SHP. Activation of pro-proliferative signaling pathways, such as the mTOR pathway, together with interruption of cell division gatekeepers, such as p21, lead to hyperplastic parathyroid glands in CKD. Disruption of the parathyroid circadian clock may also contribute to parathyroid cell proliferation in SHP. Figure 3 summarizes the complex mechanisms that contribute to the increased PTH production and parathyroid cell proliferation in CKD-induced SHP. In the lack of a functional parathyroid cell line, most studies were performed in experimental animal models that are supported by studies using human tissues. Understanding the molecular mechanisms of CKD-induced SHP may identify new intervention targets for the control of SHP.

## Abbreviations

PTH	parathyroid hormone
SHP	secondary hyperparathyroidism
CKD	chronic kidney disease
CaSR	Calcium sensing receptor
VDR	vitamin D receptor
FGF23	fibroblast growth factor 23
MiRNA	micro-RNA
MiRNome	miRNA transcriptome
3′-UTR	3′-untranslated region
ARE	AU-rich element
AUF1	adenosine-uridine (AU)-rich binding factor 1
KSRP	K-homology splicing regulatory protein
CPEB protein	cytoplasmic polyadenylation element binding protein
PT-*Dicer^−/−^*	parathyroid-specific gene deletion of *Dicer*
EGFR	epidermal growth factor receptor
Mtor	mammalian target of rapamycin
mTORC	mammalian target of rapamycin complex
TGF-α	transforming growth factor α
1:25D	1,25 dihydroxy-vitamin D
NF-kB	nuclear factor kappa B
COX	Cyclooxygenase
PGE2	prostaglandin E2
EP2	PGE2 receptor 2 subtype
FGFR	fibroblast growth factor receptor
*rpS6p^−/−^*	ribosomal protein S6 (rpS6) harboring alanine substitutions at all serine phosphorylation sites
MAPK	mitogen-activated protein kinase
PRAD1	parathyroid adenomatosis 1
MEN1	multiple endocrine neoplasia type 1
AKT	protein kinase B
